# Tractography in Curvilinear Coordinates

**DOI:** 10.3389/fnins.2021.716538

**Published:** 2021-08-26

**Authors:** Uzair Hussain, Corey A. Baron, Ali R. Khan

**Affiliations:** ^1^Centre for Functional and Metabolic Mapping, Robarts Research Institute, Western University, London, ON, Canada; ^2^Department of Medical Biophysics, Schulich School of Medicine and Dentistry, Western University, London, ON, Canada; ^3^School of Biomedical Engineering, Western University, London, ON, Canada

**Keywords:** MRI, diffusion MRI, tractography, hippocampus, curvilinear coordinates

## Abstract

Coordinate invariance of physical laws is central in physics, it grants us the freedom to express observations in coordinate systems that provide computational convenience. In the context of medical imaging there are numerous examples where departing from Cartesian to curvilinear coordinates leads to ease of visualization and simplicity, such as spherical coordinates in the brain's cortex, or universal ventricular coordinates in the heart. In this work we introduce tools that enhance the use of existing diffusion tractography approaches to utilize arbitrary coordinates. To test our method we perform simulations that gauge tractography performance by calculating the specificity and sensitivity of tracts generated from curvilinear coordinates in comparison with those generated from Cartesian coordinates, and we find that curvilinear coordinates generally show improved sensitivity and specificity compared to Cartesian. Also, as an application of our method, we show how harmonic coordinates can be used to enhance tractography for the hippocampus.

## 1. Introduction

Traditionally, analysis of diffusion MRI (dMRI) images is performed in the Cartesian coordinates that the data is acquired in. Cartesian coordinates are well-suited for problems in which there is planar symmetry or in situations where there is no apparent symmetry or preferred direction. For example, in a MRI scanner the main magnetic field has an approximate planar symmetry, and usually scanner coordinates are chosen such that the *z*-direction points in the same direction as the main magnetic field. The remaining two coordinates are set perpendicular and parallel to the ground, which is again a preferred choice as patients are oriented horizontally (Brown et al., 2014). In contrast, a problem with spherical symmetry, for example geospatial weather data, spherical coordinates would be more suitable, further, these spherical coordinates would be fixed so that the poles of the coordinates align with the axis of rotation of the Earth so that the rotation of the planet is a simple coordinate translation.

As we move our focus to specific areas within the body, the preferred directions depart from those offered by a simple Cartesian coordinate system, because most anatomical structures have complex and curved geometries for which curvilinear coordinates are more suitable. A typical example of this is the neocortex, which is a highly complicated structure due to its curved gyri and sulci. Since the microstructure of the cortex is arranged in laminae there is a preferred radial direction perpendicular to the surface of the cortex. The use of curved coordinates goes beyond just the choice of preferred directions. As shown by Bok (1929), cortical segments tend to preserve volume while the thickness of layers varies in response to the cortical folds. This observation is then used to construct an equi-volume three-dimensional coordinate system for the neocortex whose constant depth surfaces align much more accurately to observed cortical layering (Waehnert et al., 2014). Other coordinate systems have also been used on the cortex, a popular choice is the mapping of the cortex to a sphere as implemented in Freesurfer (Fischl et al., 1999). Curvilinear coordinate systems have also been devised for the heart. Universal Ventricular Coordinates are a system of four intuitive coordinates found by employing Laplace's equation (Bayer et al., 2018).

For an application of the methods developed in this study we will focus on the hippocampus, a heavily studied structure of the brain. It plays a central role in learning processes, memory, and spatial navigation. It is also involved in major disease states (Hampel et al., 2008; Thom et al., 2010; Coras et al., 2014; Dinkelacker et al., 2015). The hippocampus can be partitioned into three distinct sub-regions: the dentate gyrus (DG), the hippocampus proper, and the subiculum (Sub). The hippocampus proper consists of three subfields, called CA1, CA2, and CA3. In cross sections, the CA subfields and the DG form two interlocked “C” shapes. Macroscopically, the hippocampus has a complex curved geometry. This complex geometry and its small size makes studying the hippocampus a challenge with current *in-vivo* imaging techniques. Using Laplace's equation and appropriate boundary conditions, harmonic coordinates can be found for the hippocampus (DeKraker et al., 2018). These coordinates also allow us to virtually unfold the hippocampus, which is done by resampling data on a grid of the domain of the coordinate system (DeKraker et al., 2018).

The focus of our work will be dMRI tractography in curvilinear coordinates. Tractography is the only method for studying structural connectivity *in vivo*, and has numerous applications in neuroscientific and clinical research, including by neurosurgeons for surgical planning (Essayed et al., 2017). Thus, it is necessary that tractography be able to accurately predict the presence or absence of underlying pathways. This translates into requiring the tractography procedure to have a high sensitivity and specificity (Schilling et al., 2019). A particular strain on the accuracy of tractography methods is placed by the geometry and scale of the underlying true fibres. In typical diffusion scans the resolution of voxels is on the scale of millimeters whereas the underlying structures are at microscopic scales. In addition, fibres with crossing, kissing, fanning and fibres with high curvature have been shown to have low tractography accuracy (Leergaard et al., 2010; Tournier, 2010; Ning et al., 2015).

The goal of this work is to compare tractography performed in Cartesian coordinates with tractography performed in curvilinear coordinates whose tangents point along the direction of the underlying microscopic fibres. We are particularly interested in configurations with high curvature as these are the ones that arise in the hippocampus and the neocortex. To make this comparison we perform simulations and construct the diffusion signal generated by a family of fibres that traverse around a sharp bend (tangential fibres) and are intersected by orthogonal fibres (radial fibres). To keep our comparison simple, and make interpretation of results more straight-forward, our simulations are restricted to two dimensions and we use conformal curvilinear coordinates. Next, by seeding only the tangential region we are able to gauge the performance of both coordinates by measuring the sensitivity and specificity of the resulting tracts. We find that in almost all scenarios curvilinear coordinates show improved performance over Cartesian coordinates.

As an application of our method we show how tractography can be performed on the hippocampus with the harmonic coordinates used in DeKraker et al. (2018). It is worth mentioning that our approach is general in the following manner: in the landscape of tractography there are numerous different mathematical approaches, algorithms and implementations, however, what is common amongst them is that all these approaches have their roots in the diffusion signal and the grid it is sampled on. Hence, we demonstrate how the diffusion signal itself can be resampled on to curvilinear coordinates which allows the use of these existing tools for tractography without constraining ourselves to scanner-given Cartesian coordinates.

## 2. Methods

### 2.1. Resampling dMRI Data and the Jacobian Matrix

Let *u*^*i*^ = (*u, v, w*) be a new set of coordinates, where each *u*^*i*^ is a scalar function of *x*^*j*^ = (*x, y, z*). A scalar signal *S*(*x*^*j*^) is straightforward to transform to new coordinates, we have simply, *S*(*u*^*i*^): = *S*(*u*^*i*^(*x*^*j*^)). However, dMRI data is vector valued, i.e., for each b-vector, $\vec{b}$, we have a corresponding signal $S\left(\vec{b},x^{j}\right)$. The transformation to new coordinates is now given by, $S(\vec{b}\prime ,u^{i}):=S\left(J\vec{b},u^{i}(x^{j})\right)$, where $\vec{b}\prime =J\vec{b}$ and *J* is the Jacobian matrix,

(1) $$J_{ij}=\frac{\partial u^{i}}{\partial x^{j}}.$$

Note that this transformation means that we would, in general, have different b-vectors for each voxel in an image. This requirement can be incorporated into existing dMRI analysis pipelines by composing the Jacobian matrix to the gradient non-linear distortion corrections. These distortions tend to warp the image in a spatially dependent way and are corrected using a Jacobian matrix. Many tools allow the option to include this Jacobian matrix (usually named grad_dev.nii.gz) and can also handle the matrix for the new coordinates, making this approach quite general. If that option is not available there may still be other ways to employ the Jacobian matrix, as we show for Q-ball imaging (Tuch, 2004). Central to Q-ball imaging is the orientation distribution function (ODF), $\psi \left(\vec{b}\right)$, given by,

(2) $$\psi \left(\vec{b}\right)=\frac{1}{Z}\int_{0}^{\infty }P\left(r\vec{b}\right)dr$$

where, *P* is the ensemble average diffusion propagator and *Z* is a dimensionless constant. If tractography is performed using, ψ, the key quantity is then the directions of the peaks of ψ. Let ${\vec{v}}_{\text{peak}}$ be such a peak. To perform tractography using the new coordinates, we have to simply use the peak transformed by the Jacobian matrix, ${\vec{v}}_{\text{peak}}^{\prime }=J{\vec{v}}_{\text{peak}}$. Since only the peaks are transformed there is no effect on the angular resolution. Also, note that any function, not just the peaks, on the tangent space can be transformed this way, like the Watson distribution from NODDI (Zhang et al., 2012).

These mathematical considerations that maintain generality allow one to perform tractography in arbitrary coordinates with the insertion of a few simple steps to existing pipelines. Namely, given the existence of new coordinates in terms of scanner-given Cartesian ones, we have to perform three steps, (1) resampling the dMRI data on a grid of the new coordinates, (2) Jacobian matrix transform of the b-vectors (either we use, grad_dev.nii.gz, or transform peaks as outlined above), and (3) perform tractography on the grid of new coordinates and optionally move them back to the original grid with the inverse coordinate mapping.

### 2.2. Simulations and Conformal Coordinates

**Figure 1:**
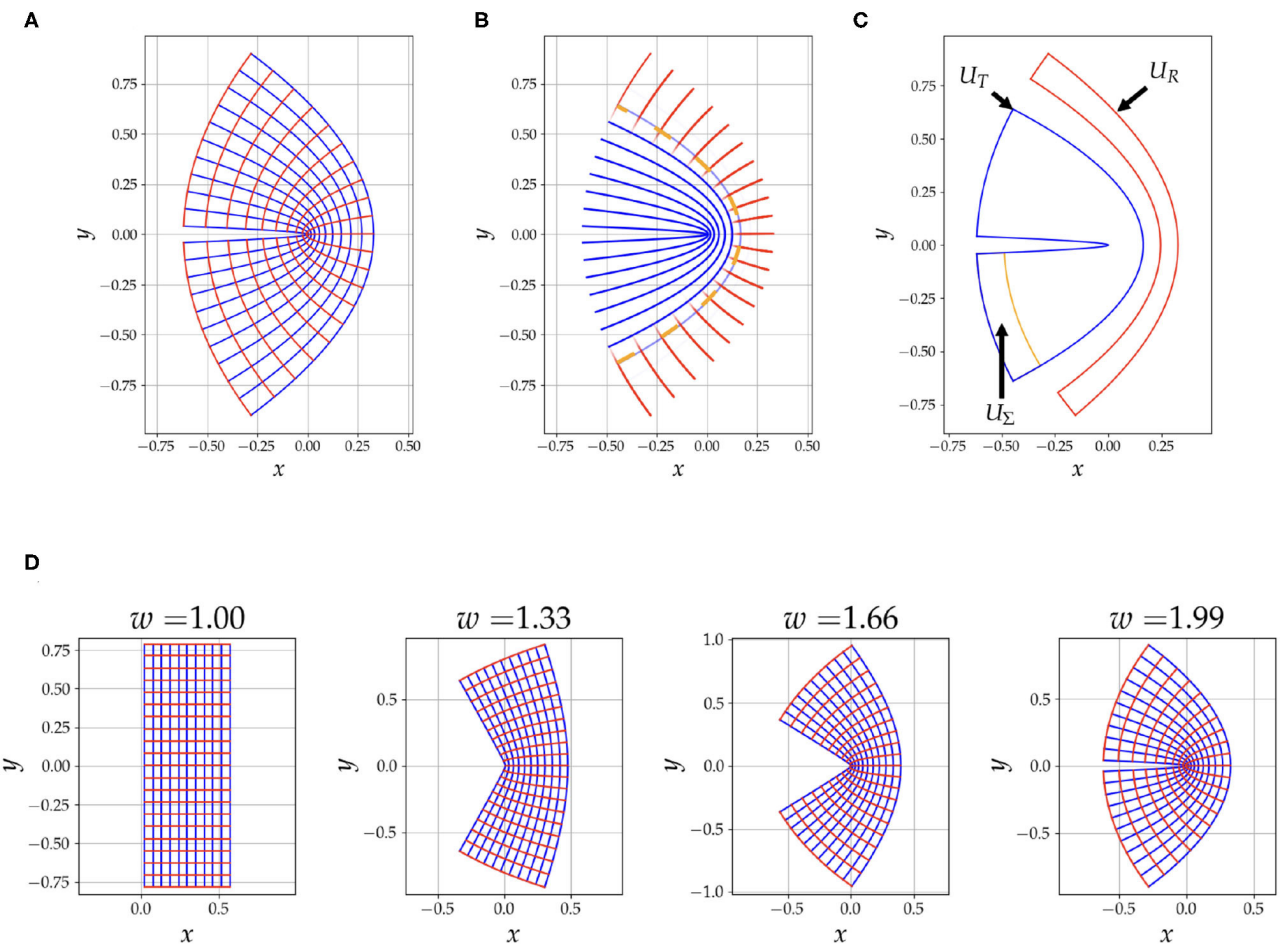
This figure summarizes aspects of our simulation strategy. In **(A)**, we have an illustration of the conformal coordinate system grid generated by Equation (3) with *w* = 1.99. In **(B)**, we have the fibres, blue for tangential and red for radial, used to generate the diffusion signal. Notice that there is an overlap between the two tracts to depict the mixing of the signal from the two compartments, the opacity of the line is calculated from Equation (4). The orange dashed line is *u*_1/2_. In **(C)**, the region *U*_Σ_, is the seeding area, the region *U*_*T*_ is the ground truth area occupied by the tangential tracts, and *U*_*R*_ is used to detect tracts that falsely traverse into the radial fibres. Notice that *U*_*R*_ is half of the region occupied by the radial tracts, this is done to reduce errors introduced by discretization. Also note that *U*_*R*_ is asymmetric, this choice is to reject false positives caused by purely radial tracts emanating from *U*_Σ_, the focus is intended to be on the bent area. In **(D)**, we show the effect of changing the parameter *w* on the curvature of the geometry.

For simplicity and ease of interpretation we restrict our diffusion simulations to two dimensions. One straightforward way to generate conformal coordinates in two dimensions is by the use of the complex plane, ℂ. Let (*u, v*) be conformal coordinates, (*x, y*) be Cartesian coordinates, and, ϕ^−1^:(*u, v*) → (*x, y*), then the conformal mapping used here is,

(3) $$\phi^{-1}=z^{w}$$

where ϕ^−1^ ∈ ℂ, *z* = *u* + *iv* and *w* ∈ ℝ. We have then, *x*(*u, v*) = ℜ(ϕ^−1^) and *y*(*u, v*) = ℑ(ϕ^−1^). The grid generated from this mapping is shown in Figure 1A, with, *u* ∈ [0.02, 0.6], *v* ∈ [−π/4, π/4] and *w* = 1.99. The *u* = *constant* curves are shown in blue and the *v* = *constant* curves are shown in red. As demonstrated in Figure 1D the parameter *w* varies the curvature of the grid.

Next we simulate a diffusion signal from these coordinates. Along with two eigenvalues, λ_*u*_ and λ_*v*_, we use the unit tangent vectors of these coordinate curves, $\vec{u}$ and $\vec{v}$, as eigenvectors to calculate a diffusion tensor, $D_{s}^{ij}$. Then, for a given b-tensor, *b*_*ij*_, we can generate a signal, $S\sim \exp\left(-\underset{ij}{\sum }b_{ij}D_{s}^{ij}\right)$. Let *x*_1/2_ = (*x*(*u*_min_, 0) + *x*(*u*_max_, 0))/2 be the midpoint of the bend at *y* = 0 and *v* = 0. We have then the curve *u* = *u*_1/2_ where *u*_1/2_ = ℜ(ϕ(*x*_1/2_, 0)). To have the signal to transition from dominant tangential eigenvectors for *u* < *u*_1/2_ to dominant radial eigenvectors for *u* > *u*_1/2_, we use a logistic windowing function, *W*(*u*) = 1/(1 + exp(−*k*(*u* − *u*_1/2_)). We generate a tangential signal, *S*_*T*_, by choosing λ_*u*_ ≫ λ_*v*_ and a radial signal, *S*_*R*_, by choosing λ_*u*_ ≪ λ_*v*_. Our final signal is then given by,

(4) $$S_{\text{total}}=(1-W(u))S_{T}+W(u)S_{R}.$$

Figure 1B shows a depiction of the fibres used to generate the signal, the opacity of the fibres is determined with Equation (4).

In our simulations we vary the following parameters, (1) resolution, (2) curvature of the bend with the *w* parameter from Equation (3) and (3) the angle threshold, θ, for tractography, which is the maximum allowed angle that a tract can turn in one step. We expect that all these parameters will greatly influence the performance of the tractography, a lower resolution would diminish performance and there should be an interplay between *w* and θ, for example, very high curvature and moderate θ would lead to tracts not being able to traverse the bend. We choose sixteen values for each of these parameters. The resolution ranges from 0.2 to 1.2 mm, and to give this a reference, the length of the grid when *w* = 1.00 is 16 mm. The curvature for the bend ranges from no bend, *w* = 1.00, to maximum bend, *w* = 1.99. The angle threshold, θ, ranges from, θ = 20° to θ = 90°. To simulate the signal we choose the *b*-values (1,000 s/mm^2^) and b-vectors of the first shell in the HCP dataset (Van Essen et al., 2012). The baseline signal, *S*_0_, is 1,000, the dominant eigenvalue is 0.01 mm^2^/s and the second eigenvalue is 0.0001 mm^2^/s. The tractography step size is a quarter of the voxel length as per EuDx recommendation (Garyfallidis, 2013).

For all the tractography performed in this work we use the Constant Solid Angle ODF Q-ball model (Aganj et al., 2010) to estimate ODF peaks and the EuDx algorithm to generate streamlines (Garyfallidis, 2013). EuDx is an Euler's method based fast and purely deterministic approach which can take as inputs model-based or model-free reconstruction algorithms, and it is robust at crossing fibres. Both of these algorithms are implemented in the dipy library (Garyfallidis et al., 2014). Note that for using our approach no modifications are needed for the EuDx algorithm itself. The seeding region for the streamlines is, *U*_Σ_, as in shown Figure 1C. The seeds are kept the same across different coordinate systems and are generated from the mask *U*_Σ_. In addition, when we vary the resolution of our simulations, the mask for the seeds, *U*_Σ_, is kept at the highest resolution, this ensures that the seeding rate is the same across changes in resolution. For resampling all the diffusion data onto the new coordinate grid we use radial basis function interpolation.

### 2.3. Sensitivity and Specificity of Tracts

To compare the tracts from the simulation obtained from using Cartesian vs. conformal coordinates we measure the sensitivity and specificity of the tracts for both approaches. The seeding region, *U*_Σ_, is labeled in Figure 1C. In the same figure, the region occupied by the tangential tracts that span *u*_0_ to *u*_1/2_, *U*_*T*_, is also labeled. We also have the radial region, *U*_*R*_, which extends radially from *u*_3/4_, to *u*_max_. The reason to choose this sub-region over the whole region containing radial fibres is to avoid biases in the specificity caused by the discritization of the underlying grid, which is used to calculate areas. Also note in Figure 1C that *U*_*R*_ is slightly asymmetric under a reflection across the *x*-axis because the region next to *U*_Σ_ is omitted. This region is omitted to keep the focus of sensitivity and specificity on the bent region; since we use a windowing function and overlap the two families, there are tracts that are purely radial that emanate from the seed region and interfere with the sensitivity and specificity measurements in the region of high curvature. We use the following formulas to calculate the sensitivity and specificity,

(5) $$\begin{array}{l} \text{ Sensitivity}=\frac{\text{Area}\left(U_{\Sigma }\cap U_{T}\right)}{\text{Area}\left(U_{T}\right)}\\ \text{Specificity}=1-\frac{\text{Area}\left(U_{\Sigma }\cap U_{R}\right)}{\text{Area}\left(U_{R}\right)}. \end{array}$$

We also calculate Youden's J-statistic, *Y* = Sensitivity + Specificity − 1. The area in the numerator is calculated by counting the voxels that tracts pass through. For example, Area(*U*_Σ_ ∩ *U*_*T*_) is the number of voxels a tract passes through such that these voxels exist in both *U*_Σ_ and *U*_*T*_, and Area(*U*_*T*_), on the other hand, is the area of the whole tangential region. Since we vary the resolution in our simulations, we choose a voxel size of 0.2 mm to calculate the area to reduce biases introduced by discretization. Occupancy (tract density) of tracts passing a voxel in a particular region is kept binary regardless of the number of tracts that pass through.

### 2.4. Hippocampus

**Figure 2:**
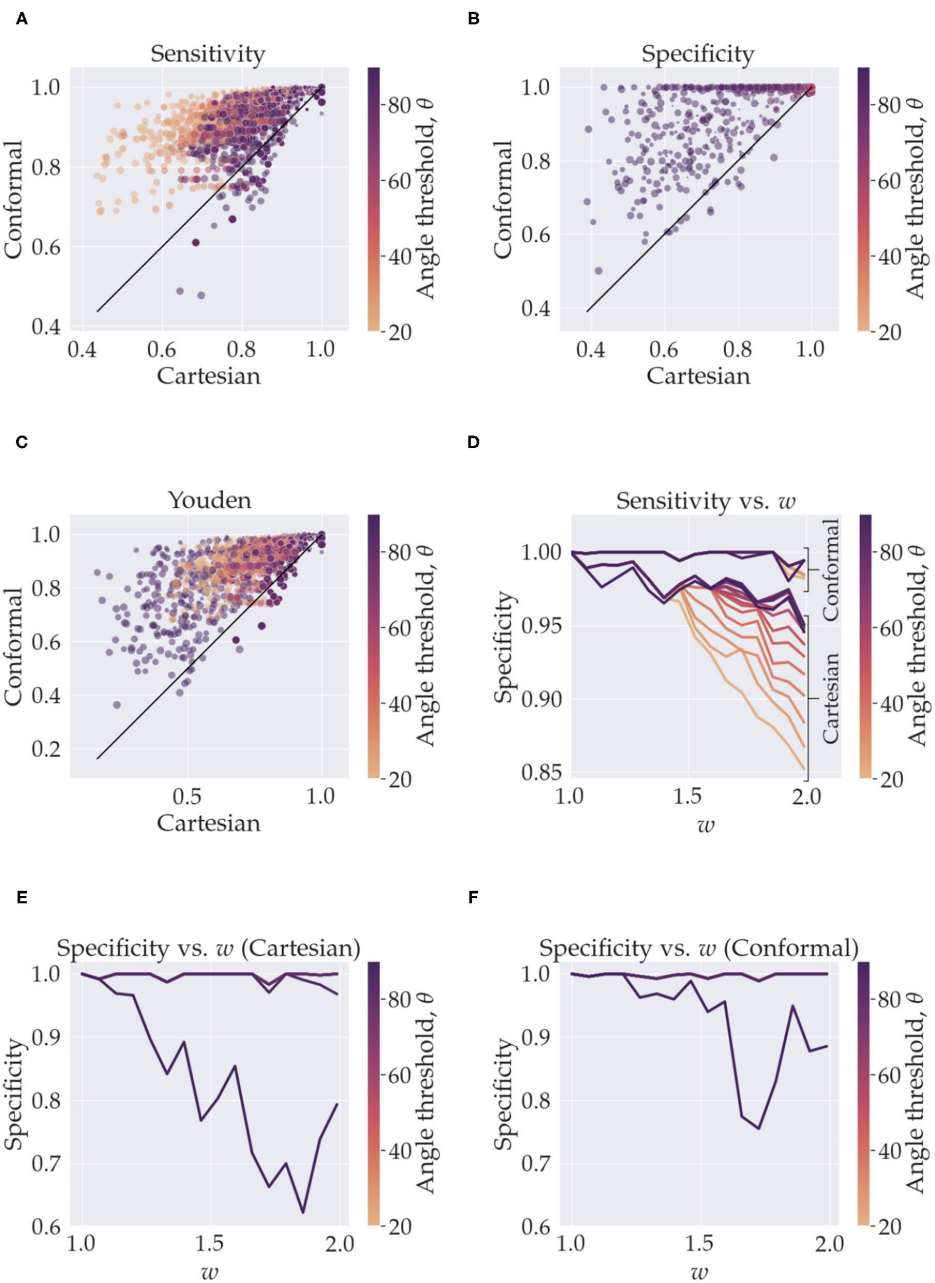
This figure summarizes the sensitivity and specificity measurements from the simulations. In **(A–C)**, the *y*-axis represents quantities calculated with using conformal coordinates and the *x*-axis represents quantities calculated with using Cartesian coordinates. The results from the whole parameter space are shown, the color of the markers represents the angle threshold, where the orange is low angles and purple is high. The size of the markers depicts the resolution, small markers are high resolution (smaller voxels) and larger markers are low resolution (larger voxels). In **(A)**, for e.g., we see that we have high sensitivity for both Cartesian and conformal coordinates when using high resolution (small markers in the top right). In **(D–F)** we isolate the effect of the angle threshold and curvature, *w*, by fixing the resolution to its maximum value. In **(D)** we see the sensitivity, for both conformal and Cartesian coordinates (labeled), as a function of the angle threshold (color) and *w* (*x*-axis). In **(E)**, we have analogous curves but for specificity when using Cartesian coordinates, and **(F)** is specificity when using conformal coordinates. In **(E)** the curves for all angles overlap except for 80° (minute deviation from angles < 80°) and for 90°. In **(F)** all curves overlap except for the 90° one.

As an application of our methods we perform tractography with harmonic coordinates on the hippocampus. We choose two random subjects (four hippocampi) from the Human Connectome Project (HCP) Young Adult 3T study, WU-Minn S1200 release (Van Essen et al., 2012). The dMRI scans are multi-shell with b-values of 0, 1,000, 2,000, and 3,000 s/mm^2^ with approximately an equal number of acquisitions on each non-zero b-shell and an echo spacing of 0.78 ms. The resolution is 1.25 mm with isotropic voxels. Manual segmentation of all hippocampi and their subfields is performed, which is followed by solving for three harmonic coordinates, *u*, *v*, and *w*, by using Laplace's equation. In Figure 2A, we see the coordinates and the boundaries chosen for each coordinate (e.g., when solving for the coordinate *u*, *U*_0_ is the source and *U*_1_ is the sink). The boundaries of these coordinates are motivated neuroanatomically, and consist of structures which border the hippocampus at its topological edge. We choose Neumann boundary conditions on the rest of the hippocampus to ensure that the tangent vectors of the coordinates are orthogonal to the surface normals at the boundary of the hippocampus. When solving for the coordinates *v* and *w*, we follow an analogous procedure (e.g., when solving for *v* we chose the source and sink as *V*_0_ and *V*_1_ respectively), and Neumann boundary conditions on the rest of the hippocampus. More details about this procedure can be found in DeKraker et al. (2018). We upsample the diffusion volumes to 0.625 mm and perform tractography in both Cartesian and harmonic coordinates. Upsampling is a standard practice in tractography. As shown in Dyrby et al. (2014), upsampling diffusion data provides more accurate geometrical information in complex regions like tract boundaries and cortical layers by reducing the partial-volume-effect, in addition, they show that upsampling also aids in tractography. The hippocampus is a small structure that is sensitive to partial volume effects, and omitting this upsampling step worsens tractography performance in both coordinate systems. The procedure used to perform the tractography is the same as the simulations, i.e, the Constant Solid Angle ODF Q-ball model (Aganj et al., 2010) to esitmate ODF peaks and the EuDx algorithm to generate tracts (Garyfallidis, 2013), the angle threshold is fixed at 60° which is the default value for EuDx in dipy (Garyfallidis et al., 2014). Using the anterior to posterior coordinate we split each hippocampus into thirds, corresponding to the head, body and tail. The tractography is done in the body of each hippocampus with step size of a quarter voxel. The seeds are in subfields CA1 and CA3, followed by filtering for tracts that pass through both subfields. Again, the seeds are kept the same across the different coordinate systems. We also compute the normalized distributions for the length and curvature of these tracts for each hippocampus.

## 3. Results

### 3.1. Simulations

**Figure 3:**
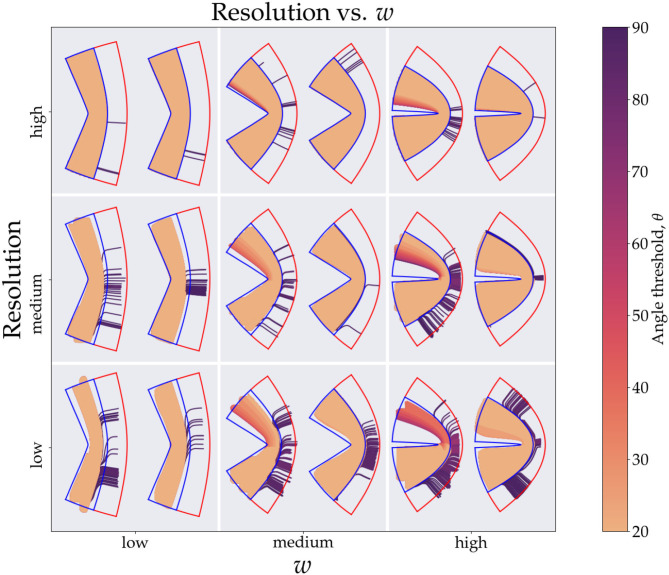
This figure shows the tracts generated from the simulations. For brevity we divide the resolution (*y*-axis) and curvature (*x*-axis) parameter space into low, medium and high values giving nine tiles. The values for the resolution are 0.2, 0.7, and 1.2 mm, respectively, and the values for the curvature are 1.24, 1.66, and 1.99, respectively. In each tile the left image is generated from using Cartesian coordinates and right one is from using conformal coordinates. The colors of the tracts represent the angle threshold used. The blue outline shows the tangential region and the red shows the radial region. Since the high angle threshold (purple) tracts occupy the most area they are plotted first and the lowest angle threshold tracts (orange) are plotted last.

**Figure 4:**
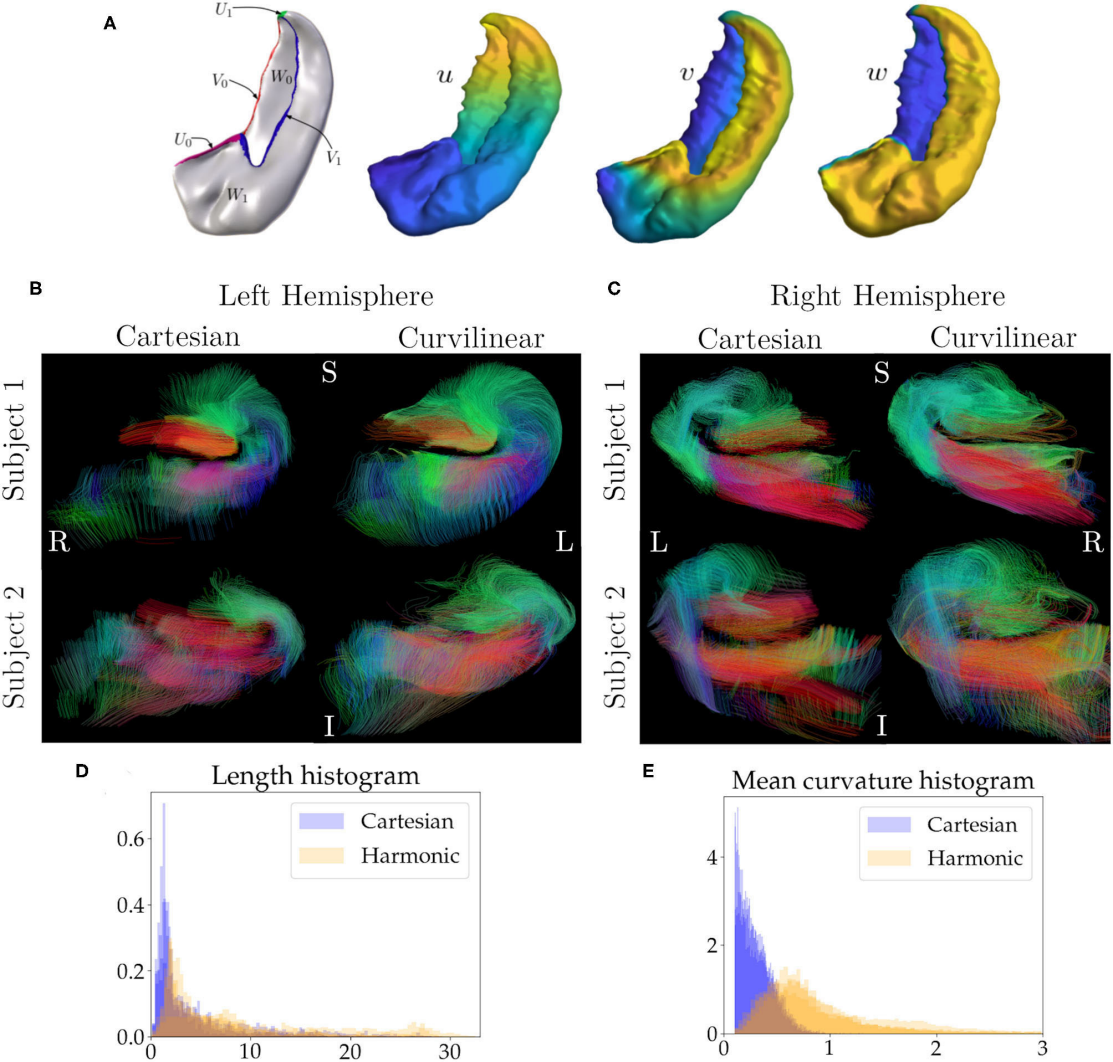
In **(A)**, we see the boundary conditions and the harmonic coordinates for the hippocampus. For example, to solve for coordinate *u*, *U*_0_ is the source and *U*_1_ is the sink, and Neumann boundary conditions are used for the rest of the hippocampus. An analogous procedure is used to compute the remaining coordinates. In **(B)** we see tracts in a coronal slice for the hippocampus of two subjects in the left hemisphere. The first column shows tracts generated from using Cartesian coordinates and the second column shows tracts generated from using curvilinear coordinates, **(C)** is analogous but for the right hemisphere. The seeds in both coordinate systems are identical. In **(B,C)**, green represents the anterior to posterior direction, red represents right to left, and blue represents inferior to superior. In **(D)**, we see a histogram for the normalized length distribution for the tracts, overlaid for each hippocampus, here blue is for Cartesian coordinates and orange is for harmonic coordinates, **(E)** is similar to **(D)** but for the mean curvature of the tracts.

In Figures 3A–C, we see the scatter plots of the sensitivity, specificity and Youden's J-statistic. The *x*-axis corresponds to measurements in Cartesian coordinates and the *y*-axis corresponds to measurements in conformal coordinates. The colors denote the angle threshold, θ and the size of the markers denote the resolution; the smaller markers represent higher resolution (smaller voxels) and larger markers represent lower resolution (larger voxels). Figure 3D shows the relationship between the sensitivity and the curvature parameter, *w*, with the colors representing the angle threshold, θ. Here, to isolate only the effects of *w* and θ, we have fixed the resolution to the smallest voxel size. There are two families of curves labeled, Cartesian and conformal, depending on which coordinate system was used. Figure 3E is similar, it shows the relationship between specificity and *w*, with colors representing θ, here, we are using Cartesian coordinates. Note that most of the θ curves overlap, except the one with θ = 90°. Figure 3F is analogous to Figure 3E but with conformal coordinates, again all angle curves overlap except 90°. Figure 4 shows resulting tracts for a reduced parameter space, we have chosen three values, low, medium and high, for the resolution (0.2, 0.7, and 1.2 mm) and *w* (1.26,1.66, and 1.99). The tracts are shaded by the angle threshold, θ. For each resolution and *w* there are two images, the left one shows the tracts generated from using Cartesian coordinates and the right one shows the tracts generated from using conformal coordinates. Also overlaid is the outline of the region with tangential tracts (blue) and the region with radial tracts (red).

### 3.2. Hippocampus

Figure 2B shows a coronal slice of the left hemisphere hippocampi tracts, generated from using Cartesian coordinates (first column) and curvilinear coordinates (second column), the anterior portion of the hippocampus is visible. Here, the tracts are colored based on their local orientation, green represents the anterior to posterior direction, red represents right to left and blue inferior to superior. Figure 2C is analogous, but for the right hemisphere. Figure 2D shows the normalized distribution for the lengths of the tracts overlaid for each hippocampus, the blue histogram is for Cartesian coordinates and orange is for harmonic. Figure 2E is a similar normalized histogram but for the mean curvature of each tract.

## 4. Discussion

In this work we explored how changing from Cartesian coordinates to curvilinear coordinates affects the performance of tractography. If our data was continuous there would be no effect of changing coordinates. But since the problem at hand is discrete we see considerable changes brought about by a change of coordinates, when the constant coordinate curves follow underlying anatomical fibres. The effect we see is that the curvilinear coordinate system indirectly informs existing tractography methods of the underlying fibres, thereby acting as an anatomical prior. Other studies have also taken similar approaches to tractography, Dong et al. (2017) used a Bayesian approach with atlas-based shape priors, Rheault et al. (2019) builds a template of streamlines, Smith et al. (2012) used image segmentation and a decision tree to anatomically constrain streamlines, and Christiaens et al. (2014) used a prior derived from 20 subjects, but to the best of our knowledge, we have not found any that use a curvilinear coordinate system to do so. Our approach is efficient in that it does not require any training data to achieve the priors and it is not derived from multiple subjects. Also, it does not filter streamline membership based on a prior atlas or a decision tree. Our method does require a segmentation of the region of interest, and an approach to find meaningful curvilinear coordinates that are generated from anatomical priors, for example, the simulations used coordinates that were generated by the curvature parameter *w* and Equation (3), and the hippocampus which used Laplace's equation with specific boundary conditions that results in coordinates that follow fibre pathways like the trisynaptic circuit. However, once these are supplied the method makes very efficient use of this information and no changes are made to existing tractography methods. Naturally, if there is no suitable candidate for a geometric prior, i.e., no strong relationship of fibres with macro-geometry or no regions of high curvature then curvilinear coordinates would not offer substantial enhancements over Cartesian coordinates. Figures 3A–C summarize the results of the simulations over the whole parameter space, and we see that using curvilinear coordinates enhances performance considerably, i.e., we measure better, sensitivity, specificity and consequently, Youden's statistic.

One of the key shortcomings of tractography done in Cartesian coordinates is in regions of high curvature (Schilling et al., 2019). To require tracts to bend in regions of high curvature the angle threshold, θ, i.e., maximum angle between two segments of the tract needs to be increased. This helps tracts turn more and increases sensitivity, but the trade-off is that now, since there is more freedom, tracts can become erratic and enter regions which are not occupied by the ground truth which lowers specificity. The limitation is even more troublesome if resolution is low in such regions of high curvature. The effect is clearly evident in our simulations. In Figure 3A, we see that, for Cartesian coordinates, the orange markers which represent low angle threshold have a lower sensitivity as opposed to the purple ones which have a higher angle threshold. The trade-off in the specificity is seen in Figure 3B where for most angle thresholds the specificity is high (overlapping points) but decreases for the purple markers. We can precisely tease out the effect of curvature and angle threshold by holding constant the diminishing effects of low resolution. Figure 3D is the plot for sensitivity vs. *w* color coded by the angle threshold, θ, with resolution fixed to its highest value. The flat family of curves are the ones resulting from using conformal coordinates. Clearly there is significant overlap in these curves which implies that the conformal coordinates tracts are invariant to the curvature, *w*. Of course, this is by design, when performing tractography on a grid of the conformal coordinates the bend does not exist, thereby vastly reducing the effects of curvature. For the Cartesian coordinates we can see the trend of diminishing sensitivity with the curvature, *w*. The drop is higher for lower angle thresholds, this is because a higher angle threshold is needed to cross the bend with increasing curvature. The least drop is caused by using a higher θ, but again the cost is specificity, as shown in Figure 3E. In Figure 3E all curves overlap except for 80° and 90°; the 80° curve is the one that deviates minutely from curves for angles <80°. Similarly in Figure 3F all curves overlap except for the 90° one. This bifurcation for the 90° curve is a direct consequence of the simulation fibres crossing orthogonally, if the crossing angle of the fibres were smaller we would observe a drop at lower angle thresholds. Notice in Figure 3F that the drop in specificity also exists for the conformal coordinates, albeit a bit less. Although, we are able to alleviate curvature effects, the radial and tangential fibres still cross at 90° when using the conformal coordinates. One aspect of note; we have not added a noise term in Equation (4), this is done to gain an understanding of the best case scenario for the two coordinate systems. We expect noise would simply decrease sensitivity and specificity for both Cartesian and conformal coordinates and not change the overall relative trends between the two approaches.

Figure 4 shows explicitly what we observe through the sensitivity and specificity metrics. We clearly see the diminishing effects of resolution, as we move down the resolution axis for a fixed curvature, less area is occupied in the tangential region (blue outline), this effect is also visible in Figure 3A, markers with smaller size which represent higher resolution have a higher corresponding sensitivity value (top right region). For the Cartesian coordinates we can explicitly see how the tracts with low angle threshold (orange) are unable to make it across the bend with the worst performance seen for lowest resolution and highest curvature (bottom right tile). We can also see the gradient of angle thresholds (colors transitioning from orange to purple) with the purple tracts being able to get around the bend the most. Note that we do not see this gradient for the conformal coordinates, again pointing toward the immunity of the conformal coordinates to high curvature.

As an application of our approach we performed tractography on four hippocampi with harmonic coordinates (DeKraker et al., 2018). In the first columns of Figures 2B,C we see the tracts generated from using the Cartesian coordinates. Notice how the hippocampus is highly curved and coronal sections closely represent our simulation scenario. The second columns of Figures 2B,C show tracts with the same seeds generated from the harmonic coordinates. We see features that resemble the outcomes from our simulations. There are regions in the Cartesian approach that have low streamline density, close to the dentate gyrus (red tracts) and also around the C-shaped bend of the hippocampus. As we do not have the ground truth, using the simulations as a guide, a reasonable assumption is that these regions have lower density, because tracts cannot overcome high curvature regions that are encountered enroute when using Cartesian coordinates. On the other hand, when using curvilinear coordinates these regions have higher density, as tracts can now overcome areas of high curvature. Notably, tracts following this C-shaped bend are expected to be present, because neuronal projections are known to connect these regions in the tri-synaptic circuit (Duvernoy, 2005); using curvilinear coordinates these connections are better represented. From Figures 2D,E, we can again see a result in line with our simulations, with harmonic coordinates the tracts are able to reach higher mean curvature values and are longer. In our simulations our coordinate tangents were aligned with the ground truth which, in practice, is difficult to achieve. Certainly for the hippocampi considered here the underlying coordinate system is not perfectly aligned with underlying fibres, yet we see better performance. In general, we expect enhancements from curvilinear coordinate systems if it has better, albeit not perfect, alignment with underlying fibres over Cartesian coordinates. When performing the tractography on the hippocampi the angle threshold was fixed to 60°. One standard way to alleviate the problem of reconstructing high curvature tracts is to increase the angle threshold, but this lowers specificity as shown by the simulations and other studies (Schilling et al., 2019). Since we do not know the ground-truth for the hippocampus to check for compromised specificity, we assume that our findings from the simulations also hold true for the hippocampus, i.e., increasing angle threshold lowers specificity and accuracy of the tracts which is remedied by using curvilinear coordinates.

One limitation of our work is that currently we are not able to mix coordinate systems, we can either perform tractography in Cartesian coordinates solely or curvilinear coordinates solely. In future work we will enhance our approach to mix coordinates systems while maintaining continuity of the tracts (and other mathematical objects), and thus are not limited to specific regions in the brain. In addition to simulations, we also demonstrated our approach in the *in vivo* hippocampus, where ground truth connectivity is not known. Here, the curvilinear approach was able to extract streamlines more consistent with anatomical expectations of connections in the tri-synaptic circuit (between subfields CA3 and CA1 of the hippocampus), recapitulating the advantages seen in the simulations. Further validation could be performed in the future as higher-resolution or histological datasets are made available.

## 5. Conclusion

In this work we have presented a novel approach to tractography that utilizes prior knowledge about fiber pathways in the form of curvilinear coordinates to enhance the sensitivity and specificity of tracts, especially in regions of high curvature and low resolution. In addition, the procedure by which we have implemented our method is general, i.e., we can take existing tractography algorithms and easily integrate our approach into them. We have shown the enhancements achieved via robust simulations where, naturally, the ground truth is known, thus laying down a solid foundation for future work regarding curvilinear coordinates and dMRI.

## Data Availability Statement

The datasets presented in this study can be found in online repositories. The names of the repository/repositories and accession number(s) can be found below: https://osf.io/94vut/, https://github.com/uhussai7/curvitract.git.

## Ethics Statement

The studies involving human participants were reviewed and approved by Western University Health Sciences Research Ethics Board. The patients/participants provided their written informed consent to participate in this study.

## Author Contributions

UH: conception, methodology, code implementation, and writing of manuscript. CB and AK: conception, methodology, and manuscript editing. All authors contributed to the article and approved the submitted version.

## Conflict of Interest

The authors declare that the research was conducted in the absence of any commercial or financial relationships that could be construed as a potential conflict of interest.

## Publisher's Note

All claims expressed in this article are solely those of the authors and do not necessarily represent those of their affiliated organizations, or those of the publisher, the editors and the reviewers. Any product that may be evaluated in this article, or claim that may be made by its manufacturer, is not guaranteed or endorsed by the publisher.

## References

[B1] Aganj I. Lenglet C. Sapiro G. Yacoub E. Ugurbil K. Harel N. (2010). Reconstruction of the orientation distribution function in single-and multiple-shell q-ball imaging within constant solid angle. Magnet. Reson. Med. 64, 554–566. 10.1002/mrm.22365 20535807 PMC2911516

[B2] Bayer J. Prassl A. J. Pashaei A. Gomez J. F. Frontera A. Neic A.. (2018). Universal ventricular coordinates: a generic framework for describing position within the heart and transferring data. Med. Image Anal. 45, 83–93. 10.1016/j.media.2018.01.005 29414438

[B3] Bok S. T. (1929). Der einflu\der in den furchen und windungen auftretenden krümmungen der gro\hirnrinde auf die rindenarchitektur. Z. Neurol. Psychiatr. 121:682. 10.1007/BF02864437

[B4] Brown R. W. Cheng Y.-C. N. Haacke E. M. Thompson M. R. Venkatesan R. (2014). Magnetic Resonance Imaging: Physical Principles and Sequence Design. Hoboken, NJ: John Wiley & Sons. 10.1002/9781118633953

[B5] Christiaens D. Reisert M. Dhollander T. Maes F. Sunaert S. Suetens P. (2014). Atlas-guided global tractography: imposing a prior on the local track orientation, in Computational Diffusion MRI, eds Bonet-Carne E. Grussu F. Ning L. Sepehrband F. Tax C. M. W. (Berlin; Heidelberg: Springer), 115–123. 10.1007/978-3-319-11182-7_11

[B6] Coras R. Milesi G. Zucca I. Mastropietro A. Scotti A. Figini M.. (2014). 7 t mri features in control human hippocampus and hippocampal sclerosis: an *ex vivo* study with histologic correlations. Epilepsia 55, 2003–2016. 10.1111/epi.12828 25366369

[B7] DeKraker J. Ferko K. M. Lau J. C. Köhler S. Khan A. R. (2018). Unfolding the hippocampus: an intrinsic coordinate system for subfield segmentations and quantitative mapping. Neuroimage 167:408–418. 10.1016/j.neuroimage.2017.11.054 29175494

[B8] Dinkelacker V. Valabregue R. Thivard L. Lehéricy S. Baulac M. Samson S.. (2015). Hippocampal-thalamic wiring in medial temporal lobe epilepsy: enhanced connectivity per hippocampal voxel. Epilepsia 56, 1217–1226. 10.1111/epi.13051 26216514

[B9] Dong X. Zhang Z. Srivastava A. (2017). Bayesian tractography using geometric shape priors. Front. Neurosci. 11:483. 10.3389/fnins.2017.00483 28936158 PMC5594407

[B10] Duvernoy H. M. (2005). The Human Hippocampus: Functional Anatomy, Vascularization and Serial Sections With MRI. Berlin; Heidelberg: Springer Science & Business Media. 10.1007/b138576

[B11] Dyrby T. B. Lundell H. Burke M. W. Reislev N. L. Paulson O. B. Ptito M.. (2014). Interpolation of diffusion weighted imaging datasets. Neuroimage 103, 202–213. 10.1016/j.neuroimage.2014.09.005 25219332

[B12] Essayed W. I. Zhang F. Unadkat P. Cosgrove G. R. Golby A. J. O'Donnell L. J. (2017). White matter tractography for neurosurgical planning: a topography-based review of the current state of the art. Neuroimage Clin. 15, 659–672. 10.1016/j.nicl.2017.06.011 28664037 PMC5480983

[B13] Fischl B. Sereno M. I. Tootell R. B. Dale A. M. (1999). High-resolution intersubject averaging and a coordinate system for the cortical surface. Hum. Brain Mapp. 8, 272–284. 10.1002/(SICI)1097-0193(1999)8:4<272::AID-HBM10>3.0.CO;2-4 10619420 PMC6873338

[B14] Garyfallidis E. (2013). Towards an accurate brain tractography (Ph.D. thesis). University of Cambridge, Cambridge, United Kingdom.

[B15] Garyfallidis E. Brett M. Amirbekian B. Rokem A. Van Der Walt S. Descoteaux M.. (2014). Dipy, a library for the analysis of diffusion mri data. Front. Neuroinform. 8:8. 10.3389/fninf.2014.00008 24600385 PMC3931231

[B16] Hampel H. Bürger K. Teipel S. J. Bokde A. L. Zetterberg H. Blennow K. (2008). Core candidate neurochemical and imaging biomarkers of Alzheimer's disease. Alzheimers Dement. 4, 38–48. 10.1016/j.jalz.2007.08.006 18631949

[B17] Leergaard T. B. White N. S. De Crespigny A. Bolstad I. D'Arceuil H. Bjaalie J. G.. (2010). Quantitative histological validation of diffusion MRI fiber orientation distributions in the rat brain. PLoS ONE 5:e8595. 10.1371/journal.pone.0008595 20062822 PMC2802592

[B18] Ning L. Laun F. Gur Y. DiBella E. V. Deslauriers-Gauthier S. Megherbi T.. (2015). Sparse reconstruction challenge for diffusion MRI: validation on a physical phantom to determine which acquisition scheme and analysis method to use? Med. Image Anal. 26, 316–331. 10.1016/j.media.2015.10.012 26606457 PMC4679726

[B19] Rheault F. St-Onge E. Sidhu J. Maier-Hein K. Tzourio-Mazoyer N. Petit L.. (2019). Bundle-specific tractography with incorporated anatomical and orientational priors. Neuroimage 186, 382–398. 10.1016/j.neuroimage.2018.11.018 30453031

[B20] Schilling K. G. Nath V. Hansen C. Parvathaneni P. Blaber J. Gao Y.. (2019). Limits to anatomical accuracy of diffusion tractography using modern approaches. Neuroimage 185, 1–11. 10.1016/j.neuroimage.2018.10.029 30317017 PMC6551229

[B21] Smith R. E. Tournier J.-D. Calamante F. Connelly A. (2012). Anatomically-constrained tractography: improved diffusion MRI streamlines tractography through effective use of anatomical information. Neuroimage 62, 1924–1938. 10.1016/j.neuroimage.2012.06.005 22705374

[B22] Thom M. Liagkouras I. Elliot K. J. Martinian L. Harkness W. McEvoy A.. (2010). Reliability of patterns of hippocampal sclerosis as predictors of postsurgical outcome. Epilepsia 51, 1801–1808. 10.1111/j.1528-1167.2010.02681.x 20738385

[B23] Tournier J. (2010). The biophysics of crossing fibers, in Diffusion MRI: Theory, Methods, and Application, ed Jones D. K. (New York, NY: Oxford University Press), 465–482. 10.1093/med/9780195369779.003.0028

[B24] Tuch D. S. (2004). Q-ball imaging. Magnet. Reson. Med. 52, 1358–1372. 10.1002/mrm.20279 15562495

[B25] Van Essen D. C. Ugurbil K. Auerbach E. Barch D. Behrens T. E. Bucholz R.. (2012). The human connectome project: a data acquisition perspective. Neuroimage 62, 2222–2231. 10.1016/j.neuroimage.2012.02.018 22366334 PMC3606888

[B26] Waehnert M. Dinse J. Weiss M. Streicher M. N. Waehnert P. Geyer S.. (2014). Anatomically motivated modeling of cortical laminae. Neuroimage 93, 210–220. 10.1016/j.neuroimage.2013.03.078 23603284

[B27] Zhang H. Schneider T. Wheeler-Kingshott C. A. Alexander D. C. (2012). Noddi: practical *in vivo* neurite orientation dispersion and density imaging of the human brain. Neuroimage 61, 1000–1016. 10.1016/j.neuroimage.2012.03.072 22484410

